# Defogging Climate Change Communication: How Cognitive Research Can Promote Effective Climate Communication

**DOI:** 10.3389/fpsyg.2016.01340

**Published:** 2016-08-31

**Authors:** Dorothee Amelung, Helen Fischer, Lenelis Kruse, Rainer Sauerborn

**Affiliations:** ^1^Department of Psychology, Heidelberg UniversityHeidelberg, Germany; ^2^Climate Change and Health Working Group, Institue of Public Health, Heidelberg UniversityHeidelberg, Germany

**Keywords:** climate change, IPCC AR5, understanding, health information, communication

It is a truism, but unfortunately not true: policy-relevant climate information should be communicated such that non-scientists can understand. Through their assessment reports, the Intergovernmental Panel on Climate Change (IPCC) periodically assesses global climate research, and creates “Summaries for Policymakers” (SPM) which constitute a synopsis of the most policy-relevant findings. The SPMs' main principles include being “audience-appropriate” and “policy-relevant but not policy-prescriptive” (IPCC, 1998). To do justice to these principles, the communicated climate science must deliver an optimal basis for decision-making by non-scientists. However, SPMs are written in a highly scientific style. In a recent linguistic analysis, SPMs' readability proved to be extremely low, scoring even below scientific publications (Barkemeyer et al., 2015). There is, however, reason to believe that more effective climate communication is possible. Recent research has shown that despite a long-held skepticism (e.g., Sterman, 2008), accessible presentation formats can increase understanding of the dynamically complex determinants of climate change (Fischer et al., 2015). Importantly, the communication of highly scientific topics such as climate change or health is generally either intended to promote predefined behavior change in the recipient (e.g., anti- smoking-campaigns; messages that aim at the reduction of red meat consumption), or may induce premature closure around specific strategies by unintentionally shaping public discourse and opinion (Amelung and Funke, 2015). Because SPMs should not be policy-prescriptive, however, they should enable informed decision-making without promoting specific response strategies or unintentionally and prematurely narrowing down public debates.

This article therefore is concerned with how to improve understanding—the accuracy of the recipient's reasoning—to allow for informed decision-making. A central aspect of improved communication is to reduce the amount of detail covered in the SPMs to a cognitively manageable degree, and to ensure that key information is conveyed. We selectively review cognitive process theories and findings derived from the area of health communication meant as starting points for empirical research toward more effective climate communication.

## Reasons for low readability: the process of writing the IPCC reports

It is crucial to know the unique IPCC editing process to understand why its readability is generally low and to propose ways to improve the communication of results to specific audiences. The following description of the editing process is based on the personal experience of one of the authors of this article (RS), who was a lead author of the most recent IPCC report's health chapter (Smith et al., 2014).

The IPCC constitutes a global panel of approximately 6000 scientists proposed by governments based on merits and geographical location. Approximately 12–15 scientists work on each chapter. A key driver of the IPCC report's low readability may be its iterative editing procedure, going back and forth between the governments, and IPCC teams. In a first stage, governments define the scope of the report, which they commission. The scientists work out three iterations of the Assessment Reports, which are rigorously evaluated—first by experts and then by the public as the pre-final drafts are made available on the internet. Everybody is allowed to comment, and review editors ensure that all comments are addressed. The third draft is then presented to a plenary of delegations from each country. This long process of evaluation and modification may adversely affect the readability of the document, because clear statements may be avoided in response to critical comments during the editing process. In such an atmosphere of extreme cautiousness, vague statements may be preferred if the scientific consensus is not unequivocal, which it rarely is.

On the other hand, the texts are also extremely detailed because each statement naturally has to be substantiated as much as possible (“traceable accounts”). As detail stands in sharp conflict with space constraints (e.g., a limit of 25 pages for all aspects related to health), texts may then be condensed into highly complex graphs, which in turn affects comprehensibility. It is therefore necessary to find communication means that limit complexity and ensure that key information is conveyed. One example area where this has been successfully achieved is health.

## Why use research on health communication as a guide for research on climate communication?

Communication on health, and climate information are similar on key aspects. First, similar to the topic of health, climate change is highly complex (Rind, 1999) due to an overwhelmingly large amount of information that grows and changes at a cognitively intractable pace. Second, both health, and climate information draw on probabilistic information that is difficult to grasp even for highly educated audiences. At the same time, probabilistic information can demonstrably be presented in ways that increase understanding (Hoffrage et al., 2000). Third, similar to the health domain, critical action needs to be taken based on uncertain information (Should we invest in adaptation? Should we invest in reforestation? e.g., Canadell and Raupach, 2008; Lobell et al., 2008). This is why it needs to be ensured that the central, decision-relevant aspects of the information is conveyed. Lastly, the climate change, and the health domains share considerable overlap in that climate change is regarded as a large and increasing health threat (Sauerborn et al., 2009; Smith et al., 2014), enhancing problems of undernutrition, injury and disease due to heat waves, and infectious diseases (Sauerborn and Ebi, 2012; Woodward et al., 2014; Phalkey et al., 2015).

However, decisions in the context of climate change may be considered more extreme than health decisions with respect to uncertainty, and decision impact. Decisions in the climate change context are characterized by large amounts of scientific uncertainty. This does not allow for unambiguous probability estimates (Amelung and Funke, 2013), even more so than in the medical field. With the communication of health/medical evidence, policy makers and the public have become accustomed to the concept of “significance.” This concept, however, can rarely be applied to climate science, further enhancing the difficulties associated with probabilistic evidence. The effects of climate change will only show decades or centuries later (Wigley, 2005), thereby also affecting future generations. The large time lags inherent in the climate system, and the large regional variability of its effects (Tebaldi and Friedlingstein, 2013) also pose particularly strong motivational barriers to take action (Gifford, 2011). Today's decision-makers will not be personally affected by the outcomes of their climate action—or inaction—, and they will not be held accountable for them, whereas decisions concerning health are typically characterized by stronger personal affection and accountability.

These extreme characteristics of climate decisions enhance the necessity of research on effective climate communication since the perceived necessity to act *per se* is lower than in health. Several years ago, it was reasoned that health communication should be more successful (World Health Organization, 2002), especially at conveying complex messages (Freimuth and Quinn, 2004), and should no longer be based on researchers' intuition but rather on evidence about how the information is understood (Reyna, 2008). A substantial amount of evidence is now available showing how effective health communication can improve understanding, quality of decision-making, health behavior, and even health itself (for reviews, see Houts et al., 2006; Coulter and Ellins, 2007; Street et al., 2009). We believe that it is high time for evidence-based (instead of constraint-based) climate communication, and that cognitive psychology can and should deliver such evidence.

## How can cognitive theory and findings from health information foster effective climate communication?

A large body of research within the cognitive sciences shows that the way information is presented affects both understanding and the credibility of the information. Specifically, analytical processing of all factual details is not sufficient or sometimes even counterproductive for comprehension of uncertain and complex information (Kahneman, 2003; Reyna, 2004; Marx et al., 2007). Highlighting the global patterns of dynamically complex systems such as the climate may lead to more accurate reasoning than a more analytical and detailed perspective (Fischer and Gonzalez, 2015). Comprehension may also be improved when the presentation format highlights key points, and helps to reduce cognitive effort (Peters et al., 2007). Moreover, studies on the effects of experienced cognitive ease demonstrate that as the difficulty of processing information increases, its estimated credibility decreases (Oppenheimer, 2006; Kahneman, 2011). This has the important implication that if readers of the SPMs experience reduced processing fluency, they may perceive the SPMs as less credible and trustworthy. In the following section, we provide some illustrations for our claim that cognitive theory and evidence could be used to enhance climate communication in the SPM's graphs and texts.

### Representation of graphs

IPCC graphs are full of detail. While this is understandable given the complexity of the topic, the wish to convey exhaustive information may exert paradoxical effects: recent meta-analyses and reviews on the communication of health information (Houts et al., 2006; Zipkin et al., 2014) find that “simple” graphs that minimize distracting details are generally better understood, so that reducing the amount of information contained in a single graph might ironically lead to better-informed decision-makers. Moreover, similarly to the results on cognitive ease mentioned above, poor comprehension of graphs seems to be associated with the impression that the evidence is of poor quality and not persuasive (Ancker and Kaufman, 2007). This has important implications also for climate change communication: if the SPM's graphs fail in comprehensibility, their messages might lose credibility and be rejected altogether.

Interestingly, when it comes to communicating interactions, data may be better understood when explained via text rather than graphs (Parrott et al., 2005; Fischer et al., 2015). Fischer et al. found that if it is explicitly mentioned how different system elements interact (such as that CO_2_ emissions and absorptions jointly shape atmospheric CO_2_ concentration), the understanding of the overall system behavior is increased, compared to the “same” information is depicted in a line graph.

### Representation of text

Due to the word-by-word negotiation cycles described above, the texts are likely to give statements that are non-controversial and factual enough that everyone can agree upon them. However, providing facts and details is not sufficient, and sometimes even counterproductive if people do not extract the appropriate *gist* (Reyna, 2008). The gist of information refers to its bottom-line, its general and qualitative meaning. Importantly, while people encode both verbatim and gist information, they prefer to operate on the crudest possible gist to make a decision, and this tendency for gist-based decisions *increases* with expertise. For example, expert cardiologists compared to medical students achieve better discrimination between higher and lower risk patients, however, the better discrimination is achieved using fewer dimensions of information (Reyna and Lloyd, 2006).

Hence, when policy-makers translate the findings presented in the SPMs into action, they will, argued from a cognitive process perspective, base their decisions on gist—and not the verbatim numbers. Such a cognitive process perspective is a useful framework for an empirical approach toward greater readability, and shows that an assessment of the representations decision-makers actually hold after reading the SPMs may serve as a crucial basis for deriving evidence-based guidelines toward greater readability. Moreover, it also does justice to the constraints of the write-up process by providing an empirical basis for the question of what information to focus on: the information that policy-makers actually rely on when making decisions.

## Conclusion

As done successfully in the area of health communication, evidence-based communication should guide the presentation of climate information. This should hold despite—or rather: because of the particular constraints posed by detailed negotiation cycles. Although the SPMs' readability was shown to be very low (Barkemeyer et al., 2015), it is yet unclear how this affects cognitive outcomes such as understanding, credibility, and use of information. We believe that cognitive research could deliver the evidence needed to communicate climate information in a way that fosters understanding and informed decision-making. Future research should therefore develop and test communication guidelines based on cognitive process theories and with appropriate target audiences such as policy-makers. Understanding of climate information is not sufficient for grounded climate action; it is however, necessary.

## Author contributions

HF and DA mainly wrote the article, RS wrote parts of the article, all authors discussed and commented on the article.

### Conflict of interest statement

The authors declare that the research was conducted in the absence of any commercial or financial relationships that could be construed as a potential conflict of interest.
